# Impact of provider specialty on the diagnosis and management of systemic lupus erythematosus in the American Indian/Alaska Native population

**DOI:** 10.1186/ar4628

**Published:** 2014-09-18

**Authors:** John A McDougall, Charles G Helmick, S Sam Lim, Caroline Gordon, Elizabeth D Ferucci

**Affiliations:** 1 Dartmouth-Hitchcock Medical Center, Lebanon, NH, USA; 2 National Center for Chronic Disease Prevention and Health Promotion, Centers for Disease Control and Prevention, Atlanta, GA, USA; 3 Emory University, Atlanta, GA, USA; 4 University of Birmingham, Edgbaston, UK; 5 Community Health Services, Alaska Native Tribal Health Consortium, Anchorage, AK, USA

## Background

Systemic lupus erythematosus (SLE) is a complex disease that is traditionally diagnosed and managed by specialists, typically rheumatologists. Higher SLE prevalence in racial/ethnic minorities such as American Indian/Alaska Native (AI/AN) people, often residing in areas with less access to rheumatologists, may necessitate diagnosis and management of SLE by primary care providers (PCP) in some cases. The purpose of this analysis was to identify areas of potential difference between PCP and specialist diagnosis and management of SLE in a population-based lupus registry of AI/AN people.

## Methods

All individuals with SLE meeting our inclusion criteria were selected from the 2009 Indian Health Service lupus registry population. Inclusion in this analysis was limited to individuals with a final diagnosis of SLE made by a PCP or specialist (dermatologist, nephrologist or rheumatologist) and documented in the medical record. Based on medical record abstraction, SLE classification criteria were validated for each individual. Testing for biologic markers of SLE and medication use at any time during the course of the disease was also abstracted.

## Results

Of the 320 patients identified with a documented physician diagnosis of SLE, 71 had been diagnosed by a PCP. SLE diagnosis by a specialist was associated with a higher median number of American College of Rheumatology (ACR) classification criteria (5 vs. 2), a higher percentage of patients meeting the definition of SLE by ACR criteria (79% vs. 22%), the Boston Weighted criteria (82% vs. 32%), and an abridged version of the Systemic Lupus International Collaborating Clinics criteria (83% vs. 35%) (*P *< 0.001 for all comparisons). Additionally, specialist diagnosis was associated with an increased proportion with any testing for anti-double-stranded DNA antibody (93% vs. 73%) and complement C3 and C4 (84% vs. 52%) documented in the medical record (*P *< 0.001 for all). Lastly, specialist diagnosis was associated with ever treatment with hydroxychloroquine (86% vs. 64%, *P *< 0.001) as documented in the medical record at any time during their disease course.

## Conclusions

Within the population studied, specialist diagnosis of SLE was associated with a higher number of SLE classification criteria met, a higher percentage of patients tested for biomarkers of disease, and a higher percentage of patients ever treated with hydroxychloroquine.

